# A Novel 3D-Printed Multi-Drive System for Synchronous Electrophysiological Recording in Multiple Brain Regions

**DOI:** 10.3389/fnins.2019.01322

**Published:** 2019-12-13

**Authors:** Jun Ma, Zifang Zhao, Shuang Cui, Feng-Yu Liu, Ming Yi, You Wan

**Affiliations:** ^1^Neuroscience Research Institute, Peking University, Beijing, China; ^2^Department of Neurobiology, School of Basic Medical Sciences, Peking University, Beijing, China; ^3^Key Laboratory for Neuroscience, Ministry of Education/National Health Commission, Peking University, Beijing, China

**Keywords:** 3D printing, electrophysiological recording, multichannel recording, neural networks, acute pain

## Abstract

Extracellular electrophysiology has been widely applied in neural network studies. Local field potentials and single-unit activities can be recorded with high-density electrodes, which facilitate the decoding of neural codes. However, the chronic multi-regional recording is still a challenging task for achieving high placement accuracy and long-term stability. Here, we present a novel electrode design with low-cost 3D-printed parts and custom printed circuits boards. This new design could facilitate precise electrode placement in multiple brain regions simultaneously and reduce the working time for surgical procedures as well. In this paper, the design and fabrication of the 3D printed multi-channel microdrive are explained in detail. We also show the result of high-quality electrophysiological recordings in eight pain-related areas from rats and the electrode placement accuracy. This novel 3D-printed multi-drive system could achieve synchronous electrophysiological recording in multiple brain regions and facilitate future neural network research.

## Introduction

To understand how our brain works, we have to observe the neural activities in different scales ranging from individual neurons to neuronal assemblies. Many techniques have been developed and adopted for these propose. Among them, extracellular electrophysiology (Ephys) excels in both spatial selectivity and temporal resolution (Buzsáki et al., 2015). Different brain areas or nuclei form a functionally bound neural circuit and support various brain functions such as memory, fear, and pain (Buzsáki, 1989; Davis, 2003; Mizuseki et al., 2009; Bastuji et al., 2016). In this point of view, it is essential to record electrophysiological activities in multiple brain areas simultaneously. However, there are several technical challenges in the long-term multi-site recording. First, the placement of the electrode requires high spatial accuracy. Signals recorded from a typical 20 μm microelectrode have a spatial reach for less than a few hundred micrometers (Buzsaki et al., 2012). Also, long-term recording requires post-implantation adjustment of electrode localization, given the negative influences on signal quality from local inflammation from the surgery, vibration and neuronal degeneration (Williams et al., 1999). Another technical challenge is the electrode implantation. It is usually challenging to achieve multi-site implantation in several sites simultaneously, because of the limited operation space on rodent’s skull, and the overall inaccuracy accumulated from individual placement errors.

The microwire-based electrode can be adapted to solve these problems. Microwires are the most basic recording device for Ephys and are still widely used nowadays. The electrodes are highly configurable: impedance can be configured by wire diameter and material (Prasad et al., 2012), whereas spatial configuration can be defined by electrode manufacturing (e.g., tetrodes) (Recce and O’keefe, 1989). Compared to nanofabricated probes, microwire-based electrodes can be easily assembled at a much lower cost. However, manually assembled electrodes suffer from spatial errors and insufficient spatial resolution. Independent movable microdrive can be used to adjust the depth of the electrode and improve placement accuracy during the experiment (Humphrey, 1970; Szabó et al., 2001; Keating and Gerstein, 2002; Battaglia et al., 2009). Most of the multi-drive systems were designed for a single implantation route. The proposed devices vary in size and weight, and the number of drives ranges from 6 to 32 (Battaglia et al., 2009; Kloosterman et al., 2009; Szabó et al., 2001; Neuralynx^1^). To target different distant brain regions, electrodes have to be able to implant in different sites/angles. Attempts have been made to record chronically from multiple brain areas with multiple independent electrodes (Nicolelis et al., 1997, 2003; Dzirasa et al., 2011). Due to the limited size of the skull, such implantation surgery is difficult, especially for small animals. It is even more challenging for multiple independent electrodes with microdrives due to the additional space occupied by the drive mechanism and the drive anchoring. Several microdrive systems have been proposed to record from multiple areas. A system modified from high-density single site drive system with 14 microdrives targeting two brain areas is designed (Lansink et al., 2007). Special designs were made to target different brain sites. In this study, a modular independent microdrive system was proposed that could record from four regions from a rat (Headley et al., 2015). In a more recent study, a 14 microdrive system was designed, but only five of them were used due to the difficulty of the implantation procedure (Billard et al., 2018). The design of the multi-site multi-microdrive system is more challenging because the location of target areas, drive mechanisms, size, and weight, ease of use needs to take into consideration and are always trading-off with each other.

Chronic recording in freely moving animals requires the implanted device to prevent the electrode from drifting due to animal moving, vibration, and other mechanical movements (Buzsáki et al., 2015). One of the most significant external applied force is coming from the pre-amp connection before the recording. It is a common practice for chronic silicon probes to have a polyimide cable to decouple the pre-amp and the electrode mechanically (Stieglitz et al., 2005; Berényi et al., 2014). However, we have not noticed that a microwire design has adopted a similar mechanism.

In recent years, 3D printing has become an affordable technology for most labs (Gross et al., 2014). Printed circuit board (PCB) manufacturing service is also straightforward to approach nowadays. Most of the 3D printers and PCB manufacturing could achieve a precision of 100 μm (Masood and Song, 2004), which could significantly increase the electrode assembly accuracy.

Here, we developed a novel design for a lightweight and reusable microwire-electrodes with modular 3D printed parts and PCBs. This design aimed to provide a device to simultaneously record multiple brain areas and reduce the workload during implantation surgery. In this design, we adopted a classic cylindric microdrive body to maximize the number of microdrives; a custom PCB board to guide multiple electrodes to their implantation sites; a flexible-PCB inter-connect to mechanically decouple the pre-amp connector and the microdrive. We have assessed the targeting accuracy, chronic recording stability of the design with the system implanted in freely moving rodents.

## Materials and Methods

### Animals

Adult male Sprague Dawley rats (280–300 g at the beginning of experiments) were provided by the Department of Laboratory Animal Sciences, Peking University Health Science Center (Beijing, China). All animals were housed in standard cages with a 12 h alternating light/dark cycle and food and water available *ad libitum*. All experimental procedures were approved by the Animal Care and Use Committee of Peking University Health Science Center and were in accordance with the Guidelines of International Association for the Study of Pain.

#### The Process of Electrode Fabrication

The explanation of the design of our system is broke down into each sub-system and organized by order of assembling.

#### Preparation of Tetrodes

Tetrodes were assembled as the previous reported (Nguyen et al., 2009). In brief, 20 μm diameter insulated Tungsten wire (California Fine Wire) was cut into a 30 cm piece and then wound together by clapping both ends of the piece with a small vessel clip which is vertically cemented to a small spin-bar. The middle of the wire then passed over a horizontally hanging rod and hooked to the vessel clip. Rotate the spin-bar with a magnetic stirrer to 10 rounds per inch. Then 210°C hot air was applied evenly to the wires for about 2 s by a reflow station. After this step, tetrode is inserted into a 75 μm inner diameter silicon capillary for mechanical support. Tetrode tip is cut with a tungsten scissor about 5 mm from the tip.

#### Design of 3D Printed Drive

**FIGURE 1:**
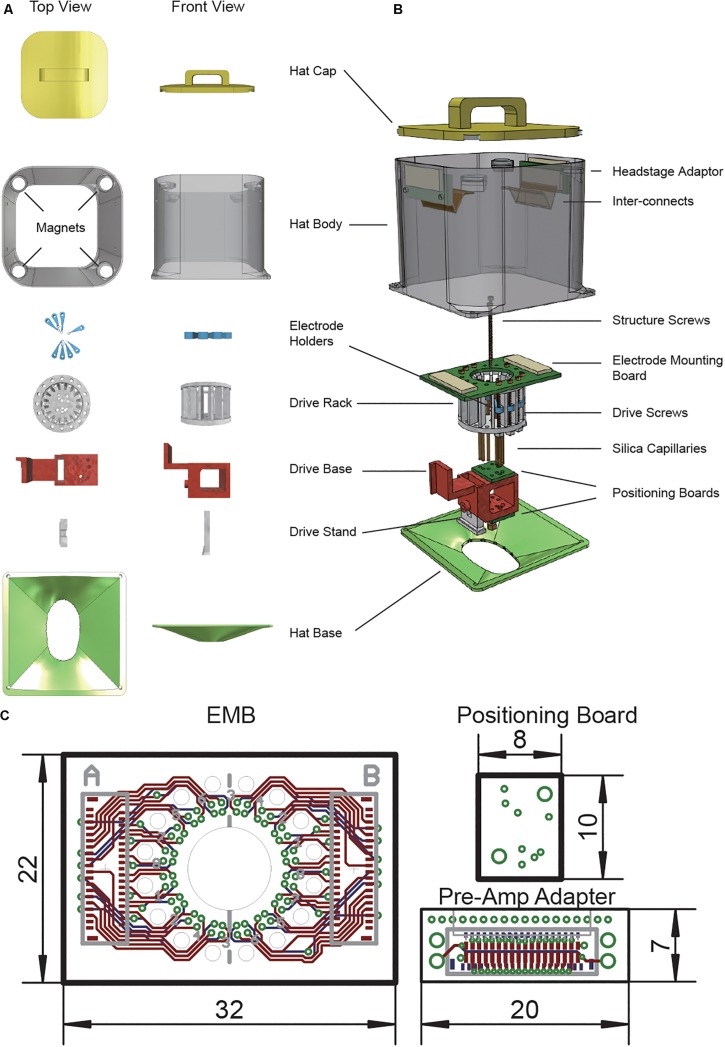
Modular high density micro-drive system design for multi-site electrophysiology. **(A)** Individual 3D printed parts used in the system. **(B)** Illustrative demonstration of a vertically disassembled system with all components. **(C)** Schematics of printed circuit boards used in this design.

We have designed a 3D printed drive to hold up to 16 independent drives (only eight were assembled in this work) and customized positioning boards that could target multiple brain areas at the same time (Figure 1) as well as a set of protective animal hat and base that protects the drive and absorb the external forces. The weight of the whole set was controlled under 15 g with 8 installed drives. 3D model was designed in 123D-Design (Autodesk Inc.).

#### Independent Movable Microdrives

Our system has a lightweight main drive rack for holding up to 16 independent movable microdrives. Each microdrive unit consists of a 2 cm long, φ1.1 mm (diameter = 1.1 mm, thread = 250 μm) brass screw, a plastic electrode holder, and a bottom nut (Figure 1 – Drive Screws, Electrode Holders). One full turn of the screw will move the microdrive for 250 μm.

#### Electrode Positioning Module

To improve the electrode implantation accuracy, we designed an electrode positioning module right below the microdrive mechanism. The positioning module consists of three parts: one drive base and two positioning boards (Figure 1 –Drive Base, Positioning boards). Positioning boards were mounted to the top side and bottom side of the drive base. Each positioning board is a custom PCB board (or laser drilled steel stencil) with the drills for all the implantation sites (Figure 1C). Two spatially separated positioning boards will limit each electrode to move with an only defined path. The bottom of the drive rack, the drive base, and positioning boards have two φ1.1 mm guideposts. Two brass screw connect all these parts with perfect alignment. The drive base also has a rectangle arm that could connect with the Stereotaxic apparatus probe holders with minimal angle misalignment. The drive base also has a rectangle opening on the bottom to connect with the replaceable drive stand.

#### Replaceable Drive Stand

We have designed a detachable drive stand to provide a secure anchoring to the scalp while easing the electrode recovery process (Figure 1 – Drive stand). Drive rack and the drive stand were fastened together by a 5 mm-long φ1.1 mm screw. When get fastened, the plastic walls of the drive base will make firm contact with the drive stand and prevent any further movement between two parts. Moreover, it is easy to recover the whole system from the skull by loosening one screw and leave the drive stand to the scalp with all the dental cement.

After the assembling of the rest of the drive body, one step before installing the electrodes, push the drive stand to the bottom slot of drive base and tighten a 5 mm-long φ1.1 mm screw to the thread in drive base.

#### Electrode Adaptor Board With Flexible Connection

Two printed circuit boards (PCB) and one flexible printed circuit (FPC) cable were designed to connect the electrode with the pre-amplifier.

A circular electrode mounting board (EMB) was designed to connect with up 64 electrodes. Each electrode is anchored to the mounting via with a small gold pin (EIB pins, Neuralynx^2^). Each group of 32 electrodes are connected to one of the FPC connectors (0.3-mm pitch, part number: 5025983993, Molex).

A pre-amplifier adapter board was designed to connect the amplifier to the electrode while anchoring to the protective hat. This board uses the same FPC connector as the EMB and has a typical pre-amplifier connector (part number: NPD-36-AA-GS, Omnetics) on the other side. Two φ1.1 mm screw holes were put on both sides of the board to anchor to the hat. The primary purpose of this part is to release the mechanic force of the pre-amplifier connection to the hat other than the drive itself.

Two boards are connected with a 5-cm long 39-contacts FPC flex cable (part number: 0150150239, Molex). The cable is made of polyimide and has a thickness of 100 μm. This part is widely applied in industry and easy to access.

All the production files of the PCB are available online^3^.

#### Protective Hat

Freely moving animals with the chronically recording setup are likely to bump their heads into the wall or objects in the environment. Another concern is the plug/unplug of the pre-amp will introduce external force to the microdrive. To keep the microdrive from moving due to those mechanical perturbations, we designed a protective hat similar to the chronic recording setup for the silicon probes (Berényi et al., 2014). The hat consists of two parts, a base to anchor to the scalp and a body as the protector for the microdrive (Figure 1- Hat body). Two holes were put near the top Hat body to mount with the pre-amplifier adapter board. We wrapped the hat with a thin layer of 65-μm copper tape then connect it to the system ground to gain extra protection against electrical noise. The hat base was designed accordingly to the rat skull. The opening of the base ensures sufficient space for the craniotomy. After the implantation surgery, the hat base is connected to the hat body by either mounting screws or superglue. Additionally, four magnets were put on top of the hat body. A 3D printed cap with another set of magnets could mount to the top to the hat body. This will protect the drive system from foreign objects when an animal is not being recorded.

### 3D Printing

A desktop FDM 3D printer (Z603S, JG Aurora) was used to perform all 3D printing. Polylactide (PLA) filament was used to build all parts. Electrode rack and drive rack were printed with a 0.05-mm slice thickness, and others used a 0.10-mm slice thickness. Printed parts were manually adjusted afterward with file and drill.

### Drive Assembling and Electrode Mounting (Figure 2)

**FIGURE 2:**
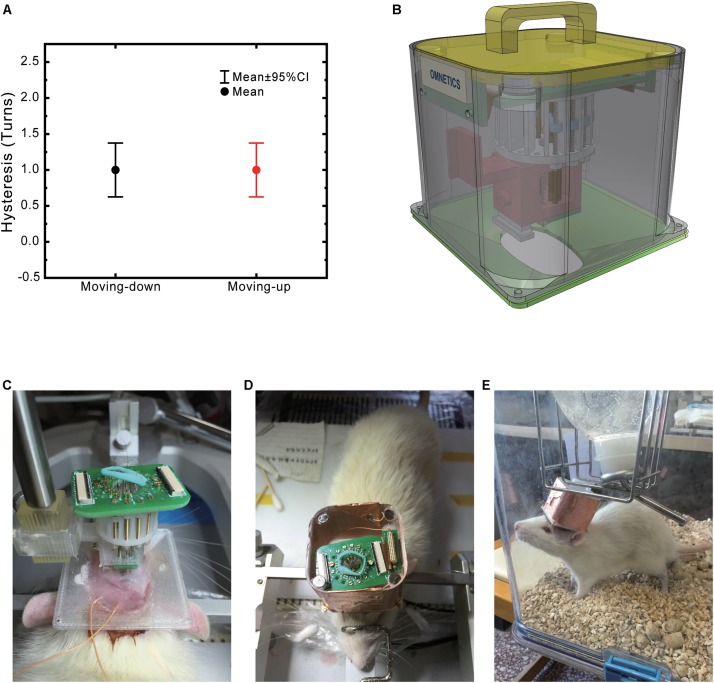
Modular drive and hat design ease implant surgery. **(A)** Micro-drive hysteresis. **(B)** 3D demonstration of an assembled system. **(C)** Implanted system during surgery. The drive stand and the cap base are fixed to the skull already. Two orange wires are connected to the ground and reference screw. **(D)** Implanted system with a complete protection hat. **(E)** Picture of a freely moving rat carrying the system.

Once the parts were printed, the drive system was manually assembled as followed:

(1) Electrode preparation: Assemble tetrodes as previously mentioned. Insert tetrodes to 3-cm long silica capillary tubes and use the superglue to fix them together by the end (microdrive side).
(2) Microdrive assembly: When assembling the microdrive, push the screw from the top end of the drive rack and thread into the electrode holders then penetrates the screw from the bottom of the rack. Fasten the nut to the bottom to make sure that there is no excess space left between the screw head or nut to the rack (to make sure the screw turning to move the microdrive instead of moving the screw). Fix the nut to the screw with super glue. Test the microdrive by turning the screw. At this point, electrode holders should move in the drive rack smoothly with the turning of the screw.
(3) Electrode positioning module: After assembling the microdrive parts, put positioning boards on both sides of the drive base, then tighten two φ1.1 mm brass screw from the bottom side of the drive base into the threads on the bottom of the drive rack. The guidepost should be aligned, and drive rack should tightly be connected with the drive base.
(4) Silica capillaries were channeled through positioning holes and glued to the electrode holder.
(5) Microwires were channeled through silica capillaries and mounted to the electrode mounting board with EIB pins. Enough length of electrode should be left between EIB and the electrode to make positive electrode are not pulling the microdrive during depth adjusting. Also, to make sure the microwire will not be damaged during the microdrive by the screwdriver. We recommend using a thin ring of plasticine to hold the excess tetrode loop in the center of the EIB board.
(6) Drive stand was connected to the bottom of the drive base with M1.1 screw and nut.
(7) In the final step, the length of each electrode was precisely measured under the microscope with the help of a laser aiming-cross that mounted to the stereotaxic apparatus. Electrode tips were trimmed and then placed to the desired length by turning the electrode drive (Figure 2).

### Recording Sites

**FIGURE 3:**
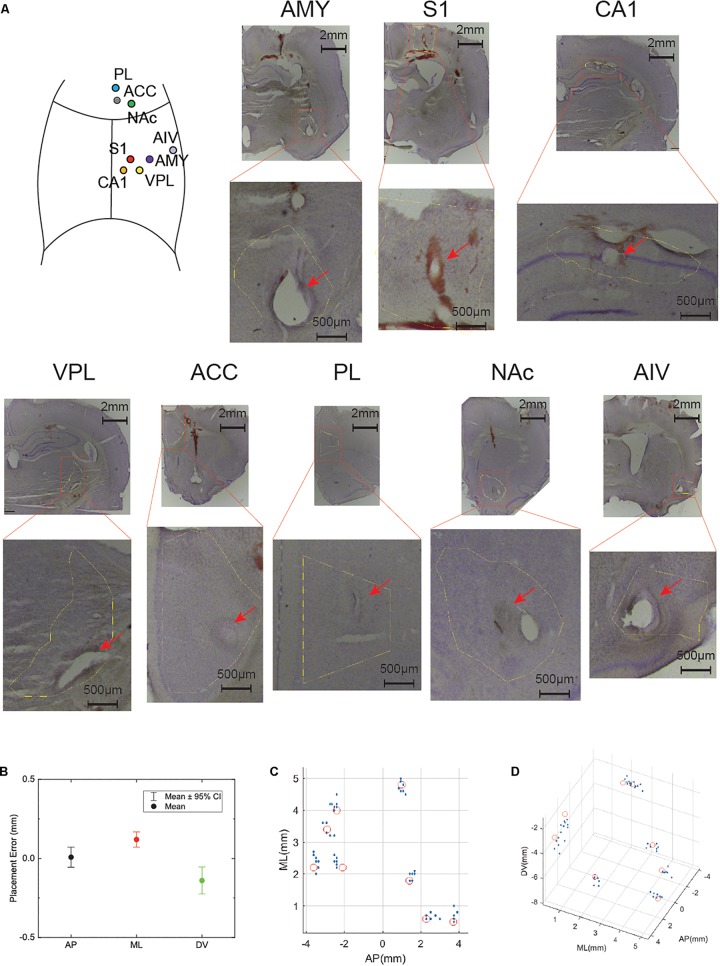
High precision electrode implantation in multiple target areas. **(A)** Histology of electrode locations in all implanted areas (Nissl staining). An illustrative implantation site map is put on the left top corner. **(B)** Statistics of implantation error in anterior-posterior (AP), medial-lateral (ML), dorsal-ventral (DV) axis. The dot shows the mean value while the bar shows 95% confidence interval (CI). **(C)** Electrode locations of all implanted rats in AP, ML axis. The red circles indicate the target location. **(D)** Electrode locations show in 3D space. The red circles indicate the target location.

This electrode was initially designed for pain-network study (Moisset and Bouhassira, 2007; Paxinos and Watson, 2009; Ploner et al., 2016). We have selected the eight brain regions that have been reported related to pain (Table 1 and Figure 3A).

**TABLE 1:** Implantation target locations.

**Target**	**Anterior-posterior**	**Medial-lateral**	**Dorsal-ventral**
Amygdala (AMY)	−2.4	4.0	−7.8
Primary somatosensory cortex (S1)	−2.1	2.2	−1.5
Hippocampus CA1 (CA1)	−3.6	2.2	−2.6
ventral posterior lateral nucleus of thalamus (VPL)	−2.9	3.4	−6.4
Anterior cingulate cortex (ACC)	2.3	0.6	−2.0
Prelimbic cortex (PL)	3.7	0.5	−3.0
Nucleus accumbens (NAc)	1.4	1.8	−7.0
ventral anterior insular cortex (AIV)	1.0	4.8	−7.4

*All units are in mm.*

### Electrode Implantation Surgery

During surgery, the rat was anesthetized with 1% sodium pentobarbital (0.5 ml/kg) and supplementary doses of less than 1/3 of the initial dose were provided when necessary to maintain anesthesia. Mannitol (20%, 5 ml/kg) was injected from the tail vein in advance to reduce intracranial pressure. The animal was head-fixed by a Kopf stereotaxic apparatus (David Kopf Instruments, Tujunga, CA, United States) and the skull was exposed. Two stainless screws were tapped into the skull in front of the recording sites with the un-pierced dura as an anchor for headset stabilization. Another two stainless screws were tapped into the skull post lambda for stabilization and served as ground and reference sites, respectively. The exposed skull was strengthened with dental Superbond (Metabond Inc.). After 3D printed hat base was cemented to the skull, craniotomies were made to expose all sites for implantation. Dura was carefully removed in all of the craniotomy sites. 3D printed drive and electrode was fixed to the stereotaxic apparatus and slowly lowered to the brain (Figure 2C). Drive stand was cemented to the skull. Gel-foam was put in all of the craniotomy sites. A 1:1 mixture of paraffin oil and paraffin (58°C melting point) was put above the gel-foam and then melted with a cauter. This will cover the implantation site without limiting the movement of the electrodes. 3D printed hat was fixed to its base through four pairs of 3 mm M1.1 screws and nuts. Ground and reference wires were connected to the amplifier adapter board in the final step (Figure 2D). The animal was allowed to rest for a week in its home cage with free access to food and water (Figure 2E).

### Electrophysiology Recording

A 32-channel electrophysiology system (RHD2000, Intan Technologies) was used to record Ephys data. Signal was sampled at 20 KHz with a 7.5 KHz low-pass filter. The frame-synchronized video was also recorded (acA1920, Basler). Drive turning was carried out by a day-by-day basis based on the numbers of recorded single-unit activities in each brain area. A step of 50 μm was used when no units were recorded, and a step of 12.5 μm was used when low signal-noise ratio spikes shown.

### Data Processing

Electrophysiology data was visualized in Neuroscope and Klusters (Hazan et al., 2006). Spike sorting was done by an automatic clustering software (Kadir et al., 2013). Manual curation was made to all automatic clustered units. We have inspected SUAs by autocorrelogram (ACG) and crosscorrelogram (CCG), noise clusters and MUA clusters are removed and clusters belong to the same unit are merged. Spectra of the LFP were computed in MATLAB with the Wavelab toolbox^4^. All the rest computation was done by custom MATLAB scripts.

Statistics of histology locations are computed in MATLAB. Electrode placement error are computed first and plotted with 95% confidential interval in Origin (OriginLab Inc.).

### Histology

We have verified the placement of the implanted electrodes and biocompatibility of the system. Histology was performed to all of the chronically implanted animals and four extra animals with acute implantation for implantation precision verification (*n* = 7). 100 μA pulses were delivered for 10 s to each recording electrode to create a marker at the recording site. The animal was then perfused with 0.9% saline, followed by 4% paraformaldehyde in 0.12 M sodium phosphate buffer (pH = 7.4). Fixative solution of 300 ml was used per 100 g of body weight. After perfusion, the animal’s brain was removed from the skull and post-fixed in the same fixative at 4°C for more than 24 h before being moved to 20% sucrose in 1 × PBS solution to dehydrate. The brain samples were then switched to 30% sucrose solution. The fixed brain was frozen by liquid nitrogen for 10 s and then cut into 50 μm thick slices. After the Nissl staining, the slices were inspected by a light field microscope.

## Results

### Multi-Site Electrode With 3D Printed Multi Independent Drive System

A lightweight 3D printed multi-channel electrode with an independent drive system was developed for multi-site micro-electrode implantation. This system consisted of six 3D-printed parts, two printed circuit boards, a flexible printed cable, and a laser-drilled stencil. Complete system weighted around 14 g (varies with drive numbers). Protective shell (Hat body, Hat base, and Hat cap) occupies 53% of the total weight. The weight of each component was as followed: drive rack 1.05 g, drive base 1.02 g, hat base 1.62 g, hat body 4.89 g, hat cap 2.34 g, screws (8 × copper screw) 0.67 g, and PCB parts 2.31 g. The weight could be further reduced by adopting a thinner PCB or replace the brass screw to titanium.

We have tested the microdrive’s movement accuracy. Drive movement hysteresis was 250 ± 45 μm (median value, standard error, *n* = 24, Figure 2A). Electrode movement were 1,250 ± 52 μm (*n* = 24) for 5 turns, 2,500 ± 50 μm (*n* = 24) for 10 turns, 3,750 ± 47 μm (*n* = 24) for 15 turns (Supplementary Figure S1).

### Precise Electrode Placement in Eight Brain Areas

We implanted eight tetrodes in eight different pain-related areas in rats. After the implantation surgery, the electrode was slowly lowered to the target depth by adjusting the microdrive. Histology showed that our electrodes were precisely implanted into the target areas (Figure 3). The overall implantation accuracy of all the recording site was 365.7 ± 28.7 μm (*n* = 56). In the next step, we checked each direction separately: 10 ± 31.7 μm for AP, 150 ± 24.1 μm for ML, −50 ± 42.7 μm for DV (Figure 3B). To better visualize the accuracy of our system, target locations and electrode locations are put together in a 3D scatter plot (Figures 3C,D).

### High-Quality Electrophysiology Data Recorded in Eight Areas

We have implanted three rats chronically with our system. All animal showed no sign of limited movement ability, and all the electrodes are functioning throughout the recording period. We started the recording once all electrodes had been lowered to the target area with a descend rate lower than 250 μm/day.

**FIGURE 4:**
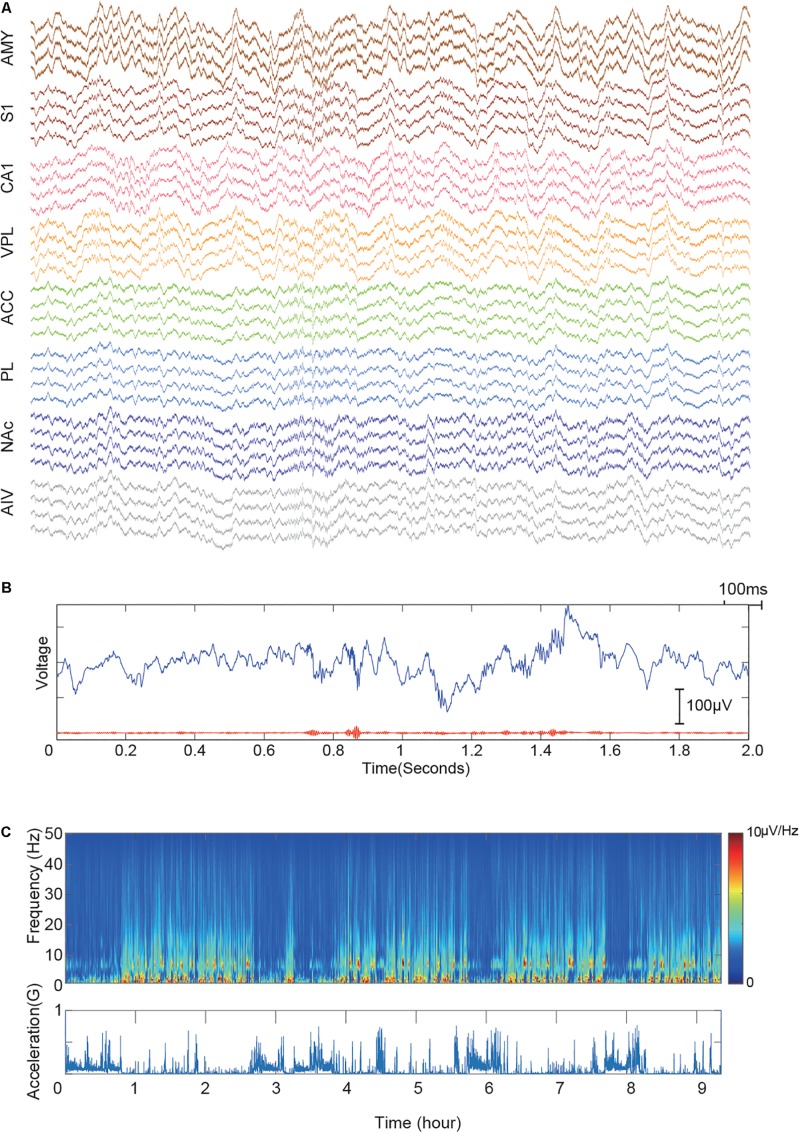
Demonstrative data showing signal quality from implanted electrodes in eight brain regions. **(A)** Simultaneously recorded raw signal trace of a 2-s window. The signal is color-grouped by brain regions. **(B)** Demonstration of recorded CA1 ripples. The upper blue trace shows raw LFP trace recorded in the hippocampal CA1 region. The bottom red line shows the 110–250 Hz bandpass filtered signal. **(C)** LFP recording quality of a 10-hour session. The upper panel shows the LFP spectra of CA1. Acceleration recorded by the onboard accelerometer is shown in the bottom panel. Multiple sleep cycles are visible from the recording.

We performed multiple long recordings (>8 h) to check the stability of the system. First, we checked the LFP recording quality. Figure 4A shows a recording sample during a sleep recording session. Prominent ripple activities in the CA1 region were observed (Figure 4B), indicating that the electrode was precisely placed in the pyramidal layer of the hippocampal CA1 region (Buzsáki, 2015). Figure 4C shows the spectra of one of the long recording sessions. The recording shows our system has very low movement-related noise and free of 50-Hz power-line noises.

**FIGURE 5:**
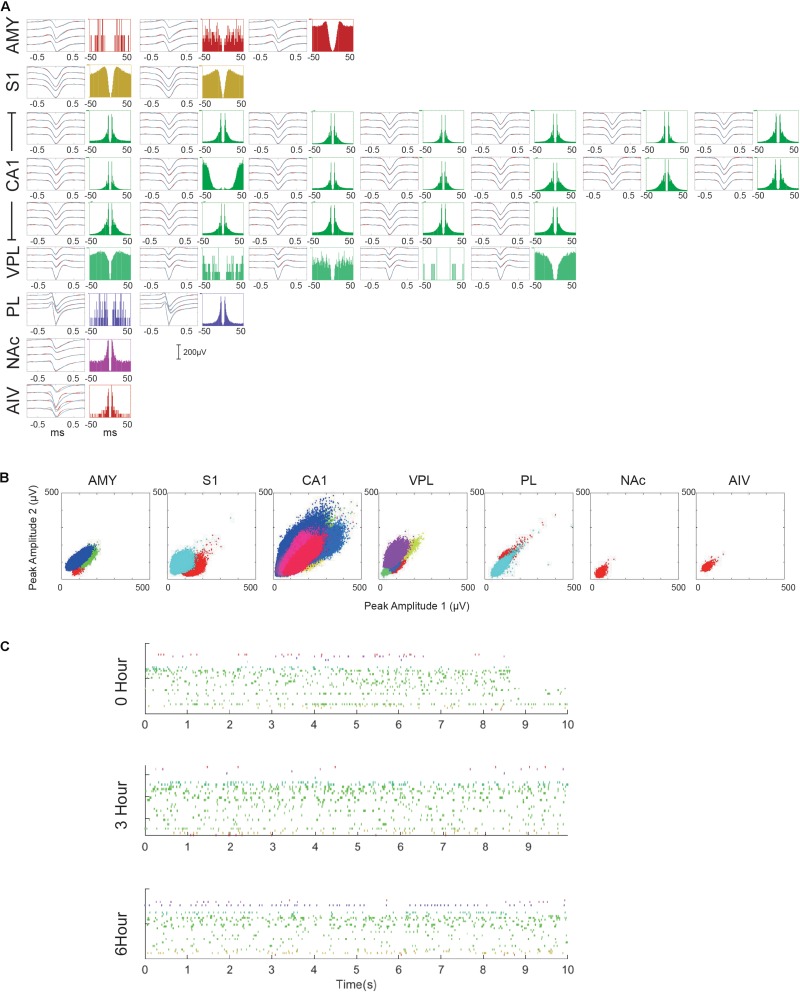
Multiple single-unit activities recorded in the 10-hour recording session. **(A)** Characters of each recorded SUA. For each SUA, the left panel shows the averaged waveform of the SUA within 0–3 h (black), 3–6 h (blue), and 6–9 h (cyan) period. Standard errors are plotted in red dashed lines. The right panel shows the auto-correlogram of each SUA. **(B)** Maximal spike amplitude of different single unit clusters over two channels. **(C)** Rasterplot of all the recorded SUA in three different periods. Only the first 10 s of each period are plotted.

Multiple single-unit activities (SUAs) were successfully recorded in all implanted regions. Low firing rate neurons (<5 spikes per minute) were excluded from the analysis. However, it is challenging to get SUA in all the areas simultaneously due to the turn-over time of recursive microdrive adjusting and verification. However, most of the recordings contained SUA from multiple brain regions. Figure 5 shows one of the best recording session. Among 9-hour recording period, 33 SUAs were recorded. In total, 355 single units were recorded over 7 days in three animals. The unit numbers of each brain region were as followed: AMY: 32, S1: 37, CA1: 218, VPL: 17, ACC: 14, PL: 13, NAc: 23, and AIV: 1. Multiple units with low firing rate were found in AIV but were not included in the unit count. Detailed spike amplitude characters are shown in Supplementary Figure S2.

### Noxious Laser-Triggered Response in Pain Network

**FIGURE 6:**
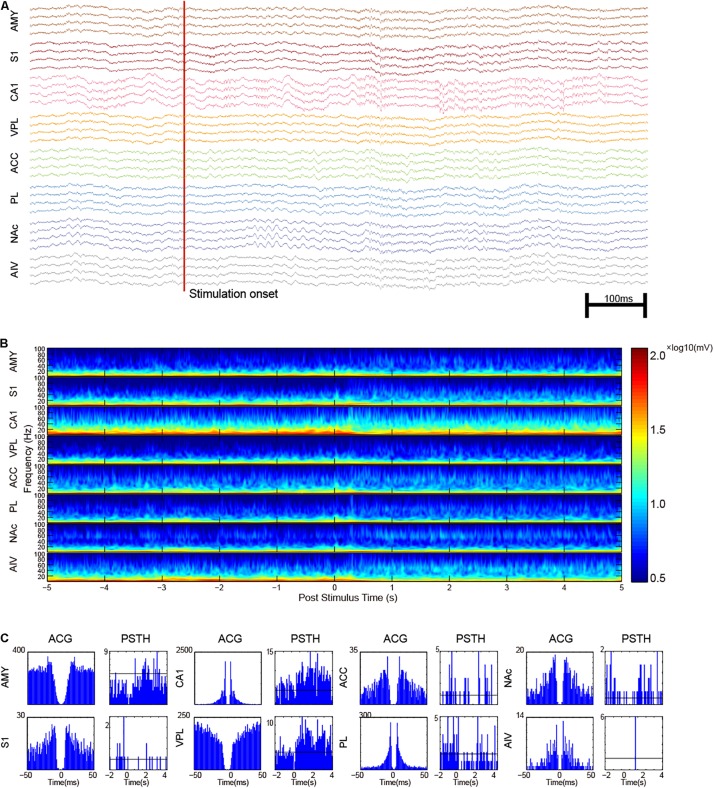
Multi-regional electrophysiology recording shows the dynamics of noxious laser evoked-response. **(A)** An example recording of a noxious laser trial with paw lifting. The red vertical line indicates the laser onset time. Increased gamma oscillation in NAc and increased spike rate in CA1 could be observed. **(B)** Trial-averaged LFP characters of recorded brain areas. Increased gamma activities were observed. **(C)** Representative SUAs in the recorded areas. Auto-correlogram (ACG) was displayed on left panels with post-stimulus time histogram (PSTH) on the other side. Neurons in AIV generally had a low firing rate.

Single-unit activities of noxious laser-evoked responses were recorded and analyzed (Figure 6A). The trial-averaged raw-waveform was not suitable for the analysis of the evoked oscillatory LFPs, so we analyzed the trial-averaged wavelet instead (Figure 6B). An increase of gamma-band oscillation was observed in CA1, ACC, NAc, and AIV. A gamma epoch in NAc was also found as shown in Figure 6A. Post-stimulation time histogram of SUAs was analyzed, and an example of noxious-stimulation responsive units in each recording site was shown in Figure 6C.

## Discussion

We have designed a 3D print multi-system drive system with high-quality Ephys data recorded from up to eight different brain areas. Our system provides the ability to record chronically from up to 16 independent adjustable tetrodes targeting different brain regions (only eight were assembled in this work). It adopts a modular design that consists of multiple 3D printed parts, which significantly reduces the manual work needed for the electrode assembling and ease the implantation process. Machine controlled part fabrication much improves the accuracy of the electrode placement. Independent movable micro-drives provide the ability to adjust the electrode depth chronically and maximize the yield of SUAs. The adoption of flexible connectors improves mechanical stability and long-term recording quality. We can successfully implant tetrodes into eight spatially separated pain-related regions. Implantation accuracy is validated in seven rats. Clean SUAs were recorded from all target areas.

### The Benefit of Multi-System Electrophysiology

Brain’s building block is functionally connected as neural assemblies. Most of the brain functions involve the interplay between several brain areas. For example, memory consolidation involves the collaboration between the hippocampus and the medial prefrontal cortex. Some of the brain function involves more brain areas. For example, more than eight different brain regions are thought to take part in the neural process of pain (Melzack and Casey, 1968; Ploner et al., 2016). To answer the question about the neural activity on a brain-wide scale, imaging-based approaches like functional magnetic resonance imaging (fMRI) and immunofluorescence could take the snapshot of the neural activity over a large spatial extent. However, those methods do not have an action potential level temporal resolution. Therefore, it is hard for those techniques to capture the fast interplays between different brain regions. Multi-site electrophysiology is especially useful for research on neural network dynamics. Till now, many high-density micro-fabricated electrodes have been developed for this purpose (Khodagholy et al., 2015; Jun et al., 2017). However, although those probes have a state of art precision and electrode density, they have certain limitations. Firstly, due to the need for a clean-room facility, most micro-fabricated electrodes are expensive. Secondly, they have certain geometry limitations. Michigan probes are etched from the silicon wafer. Therefore, all electrodes have to be limited in the same plane. High-density surface electrocorticography (ECoG) could cover a large area of the cortex but is limited to the superficial layers of cortex (Khodagholy et al., 2015). The soft material based probe could be placed in several regions at the same time, but it needs a unique implantation tool and also suffers from placement error (Zhao et al., 2019). The micro-wire based electrode has been used for neuroscience research for over 40 years (Strumwasser, 1958; O’Keefe and Bouma, 1969), and its life cycle has not come to an end yet. Many attempts have been made to use microwires to record from multiple brain areas. However, due to the limited spatial resolution, it is crucial to be able to adjust the depth of the electrode continuously after the implantation. Many investigators implanted multiple individual microdrives (Connell et al., 2013; Patel et al., 2013). A continuous effort has been put to the improved design of the microdrive system to improve the microwire implantation. Battaglia et al. (2009) introduced a less-than-2 g microdrive with six drives. There are several works adopted similar cylindrical design to hold maximal number of microdrives (Siegle et al., 2011; Freedman et al., 2016; Liang et al., 2017) and also capable of recording from a few brain regions (Lansink et al., 2007).

Moreover, a few novel designs were introduced dedicated to recording from multiple brain regions. Headley et al. (2015) come up with a modular solution to move individual electrode groups independently in different locations. Billard et al. (2018) developed a multisite microdrive dedicated to recording from multiple brain regions.

Compared to the previous works, our drive’s major difference here is the adoption of a position board to keep the implantation process easy while maintaining a high implantation accuracy. Other modifications like the mechanism to separate the pre-amplification from the microdrive were also introduced. We have shown the quality of the signal unit recording in all recorded brain areas. A simple laser-evoked pain diagram was used to show the laser-evoked responses in all recorded regions. The results showed that our design is a powerful tool to study the complex neural dynamics involving multiple distant brain regions like pain. With this tool, we can analyze the correlation between SUA/between SUA and LFP/between LFP in multiple brain regions. This would significantly increase the feature space of the analysis and leading to more findings of how neural assembly interplays with each other.

### Comparison With Other Micro-Drive Systems and Further Possible Improvements

There are several successful multi-drive systems developed. HyperDrive (Brunetti et al., 2014), Omni-drive (Neuralynx^5^) are both designed with a cylindrical shape. We adopted similar microdrive holding systems to ensure a large number of microdrives with a relatively small form factor. However, the main difference in our system is the electrode targeting mechanism. Instead of aiming at only one target, our system could have a set of custom locations. Also, compare to the systemDrive (Billard et al., 2018), no individual cannula needs to be pre-implanted for our system, and all electrodes could be implanted at once. This would require much less time for implantation surgery. Another benefit is the chance that the electrode gets kinked is almost zero.

Our design is also the first one to introduce the flexible interconnect between the pre-amplifier connector and the microdrive in the tetrode system. This design has removed one of the strongest external forces applied to the drive. During the recording, no any signal changes due to the daily plug/unplug of pre-amplification were observed.

The only chance that a large external force will apply to the microdrive itself left is the microdrive adjustment. We have trained the rat to eat fruit-loops while we were adjusting the screw on top of the head with a screwdriver. Sometimes the animal’s movement increased the force we applied to the one microdrive, and we observed signal change in other microdrives. To solve this problem, we should try to avoid excess force applying to the microdrive and, if necessary, anesthetize the animal. We also come up with an add-on device that could turn the microdrive by micro-steppers (Supplementary Figure S3). Eight micro-steppers were mounted to the same rack and installed on the top of the microdrive. We also designed a multidrive controller board that received the command from a micro-USB and turns all electrodes. However, this design would add an extra ∼10 g weight to the system. The performance of this electronic microdrive is still under validation and not included in the present study.

This probe can be further improved or modified. One direction is the integration of optogenetics parts. Optical fiber could be attached to the microdrive instead of the tetrodes. This would enable a flexible configuration of the recording/optogenetic stimulation in multiple target areas.

### Limitations

We designed the EIB board and microdrive for support up to record from 16 tetrodes, but in this study, we only showed data collected with eight tetrodes. Sixteen tetrodes will add more weight (<2 g) to the previously reported weight by eight brass screws and one other adapter board. More assembly time and micro-drive adjusting time would be expected for 16 tetrodes setup.

## Conclusion

To conclude, our design is an easy solution for electrophysiology recording in multiple spatially separated brain regions in a freely moving rat. All the parts for the drive system could be 3D-printed or purchased. The modular design of our system also eases the electrode assembling process and reduces the workload for the implantation surgery. Histology shows the high precision and repeatability of the electrode placement. High-quality LFP and single-unit activities from all the targeted areas have been recorded.

## Data Availability Statement

The raw data supporting the conclusions of this article will be made available by the authors, without undue reservation, to any qualified researcher.

## Ethics Statement

All experimental procedures were approved by the Animal Care and Use Committee of Peking University Health Science Center and were in accordance with the Guidelines of International Association for the Study of Pain.

## Author Contributions

JM, ZZ, and YW designed the experiment. JM, ZZ, SC, and F-YL performed the experiment. JM and ZZ analyzed the data. ZZ, JM, MY, and YW wrote the manuscript.

## Conflict of Interest

The authors declare that the research was conducted in the absence of any commercial or financial relationships that could be construed as a potential conflict of interest.

## Footnotes

1 https://neuralynx.com/hardware/halo-18-microdrive

2 https://neuralynx.com/hardware/halo-18-microdrive

3 https://github.com/PKU-Wanlab/TetrodeDrive

4 http://statweb.stanford.edu/~wavelab/

5 https://neuralynx.com/hardware/halo-18-microdrive

## Supplementary Material

FIGURE S1 Microdrive adjustment error measurement. **(A)** Measuring microdrive movement error under stereotaxis. An aiming laser was used for precise adjustment/measurement of electrode length. **(B)** Electrode movement error (*n* = 24).

FIGURE S2 Spike characters of long-term recording. **(A)** Maximal spike amplitude of different single unit clusters across different channels. **(B)** PCA clustering for the recorded single unit. Only two principal components were plotted. Only five CA1 units are plotted to make a better visualization.

FIGURE S3 Microdrive system with stepper control. **(A)** Custom designed eight motor controller boards. **(B)** Microdrive system with add-on stepper motors. **(C)** Simultaneously recording and microdrive adjustion. **(D)** Control interface example of each individual stepper motors.
