# Supplementary figures and images for: A Novel 3D-Printed Multi-Drive System for Synchronous Electrophysiological Recording in Multiple Brain Regions
Source: Front Neurosci. 2019 Dec 13;13:1322. doi: 10.3389/fnins.2019.01322 (PMC6923182; doi:10.3389/fnins.2019.01322)

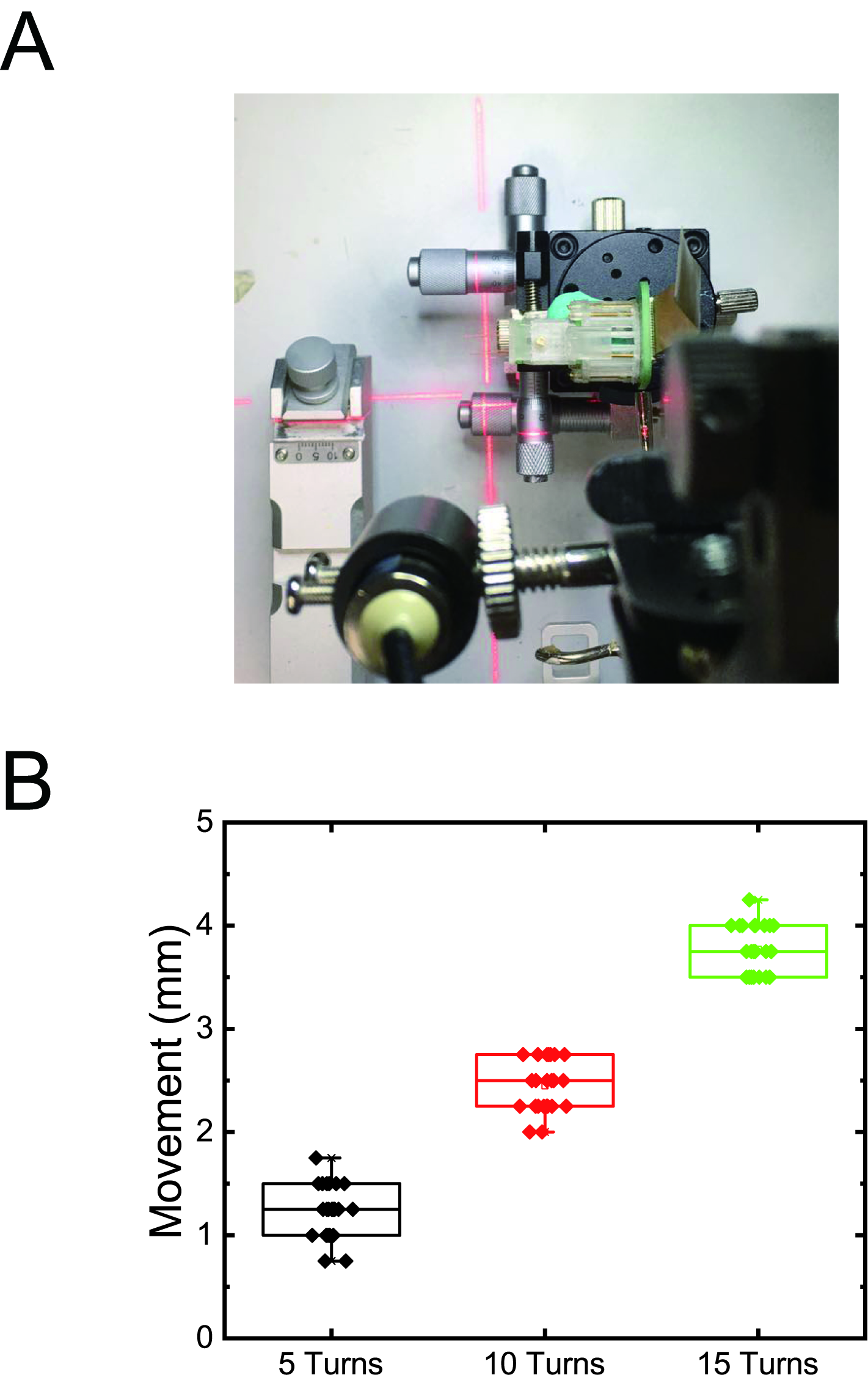

Supplement: FIGURE S1 — Microdrive adjustment error measurement. (A) Measuring microdrive movement error under stereotaxis. An aiming laser was used for precise adjustment/measurement of electrode length. (B) Electrode movement error (n = 24). [file Image_1.TIF]

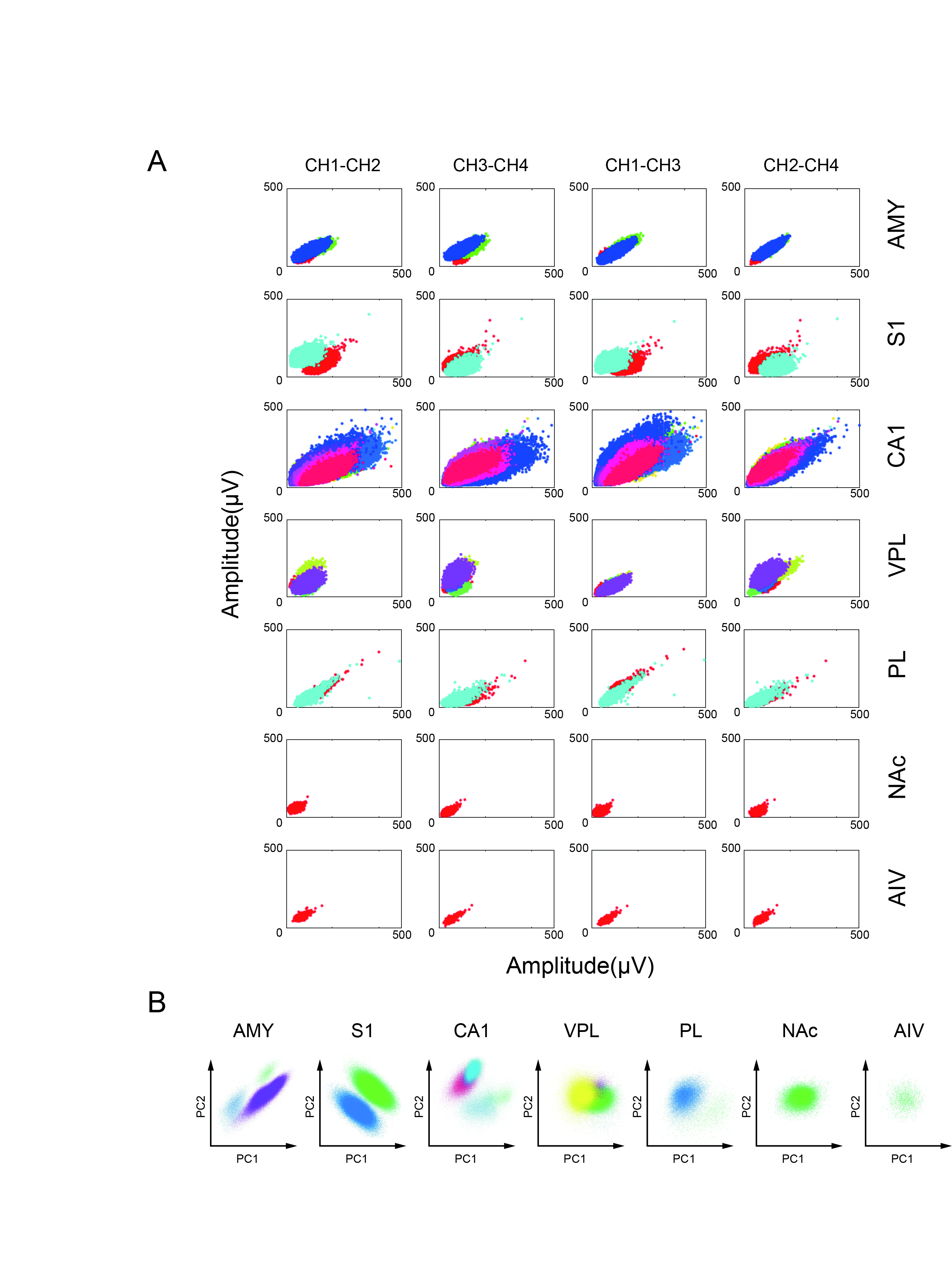

Supplement: FIGURE S2 — Spike characters of long-term recording. (A) Maximal spike amplitude of different single unit clusters across different channels. (B) PCA clustering for the recorded single unit. Only two principal components were plotted. Only five CA1 units are plotted to make a better visualization. [file Image_2.TIF]

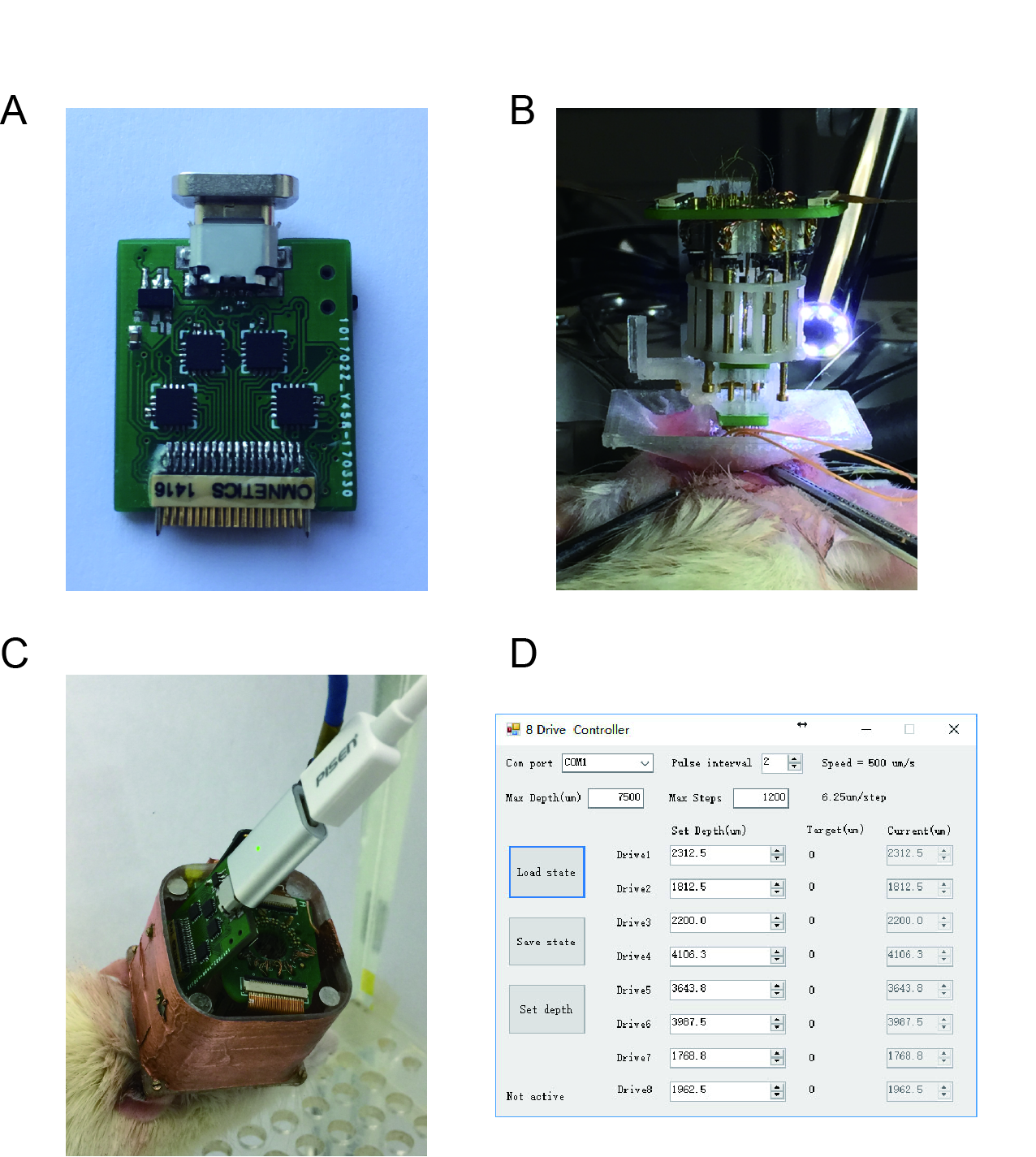

Supplement: FIGURE S3 — Microdrive system with stepper control. (A) Custom designed eight motor controller boards. (B) Microdrive system with add-on stepper motors. (C) Simultaneously recording and microdrive adjustion. (D) Control interface example of each individual stepper motors. [file Image_3.TIF]
